# HDBStat!: A platform-independent software suite for statistical analysis of high dimensional biology data

**DOI:** 10.1186/1471-2105-6-86

**Published:** 2005-04-06

**Authors:** Prinal Trivedi, Jode W Edwards, Jelai Wang, Gary L Gadbury, Vinodh Srinivasasainagendra, Stanislav O Zakharkin, Kyoungmi Kim, Tapan Mehta, Jacob PL Brand, Amit Patki, Grier P Page, David B Allison

**Affiliations:** 1 Section on Statistical Genetics, Department of Biostatistics, University of Alabama at Birmingham, Birmingham, AL 35294, USA; 2 Department of Mathematics and Statistics, University of Missouri-Rolla, Rolla, MO 65409, USA; 3 Department of Agronomy, Iowa State University, Ames, IA 50011, USA; 4 Pennington Biomedical Research Center, 6400 Perkins Rd., Baton Rouge, LA 70808, USA

## Abstract

**Background:**

Many efforts in microarray data analysis are focused on providing tools and methods for the qualitative analysis of microarray data. HDBStat! (High-Dimensional Biology-Statistics) is a software package designed for analysis of high dimensional biology data such as microarray data. It was initially developed for the analysis of microarray gene expression data, but it can also be used for some applications in proteomics and other aspects of genomics. HDBStat! provides statisticians and biologists a flexible and easy-to-use interface to analyze complex microarray data using a variety of methods for data preprocessing, quality control analysis and hypothesis testing.

**Results:**

Results generated from data preprocessing methods, quality control analysis and hypothesis testing methods are output in the form of Excel CSV tables, graphs and an Html report summarizing data analysis.

**Conclusion:**

HDBStat! is a platform-independent software that is freely available to academic institutions and non-profit organizations. It can be downloaded from our website .

## Background

One of the most critical tasks in the field of biology is identifying how and which genes interact with each other under different conditions. Until a few years ago, researchers were only able to accomplish this task for a limited number of genes because the traditional methods in molecular biology allowed them to assess only one gene at a time. The advent of microarray technology has provided investigators the opportunity to simultaneously assess the expression levels of thousands of genes. Microarrays also generate a large amount of data in short period of time. Extracting statistically valid and biologically relevant information from such massive data sets is a major challenge. HDBStat! is a user-friendly and platform-independent software designed for the statistical analysis of microarray data using well-validated methods for quality control of experiments and the identification of differentially expressed genes.

## Implementation

**Figure 1:**
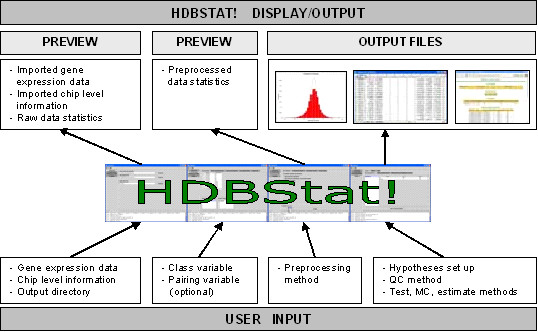
Data analysis in HDBStat! is divided into four steps – data import, data preprocessing, quality control and hypotheses testing. At each step, user input is required and in return, the results are displayed in the interface and/or output to a file.

Data analysis in HDBStat! is divided into four steps – data import, data processing, quality control and hypotheses testing (Figure 1).

### Data import

**Figure 2:**
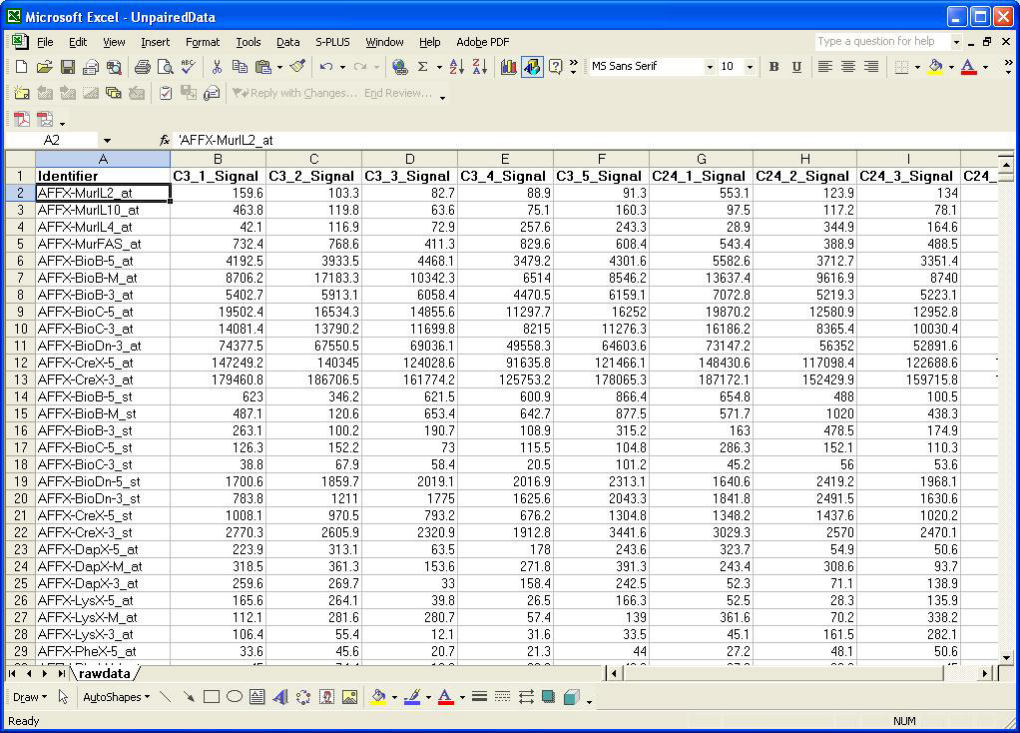
Screenshot of gene expression data file in Excel format

**Figure 3:**
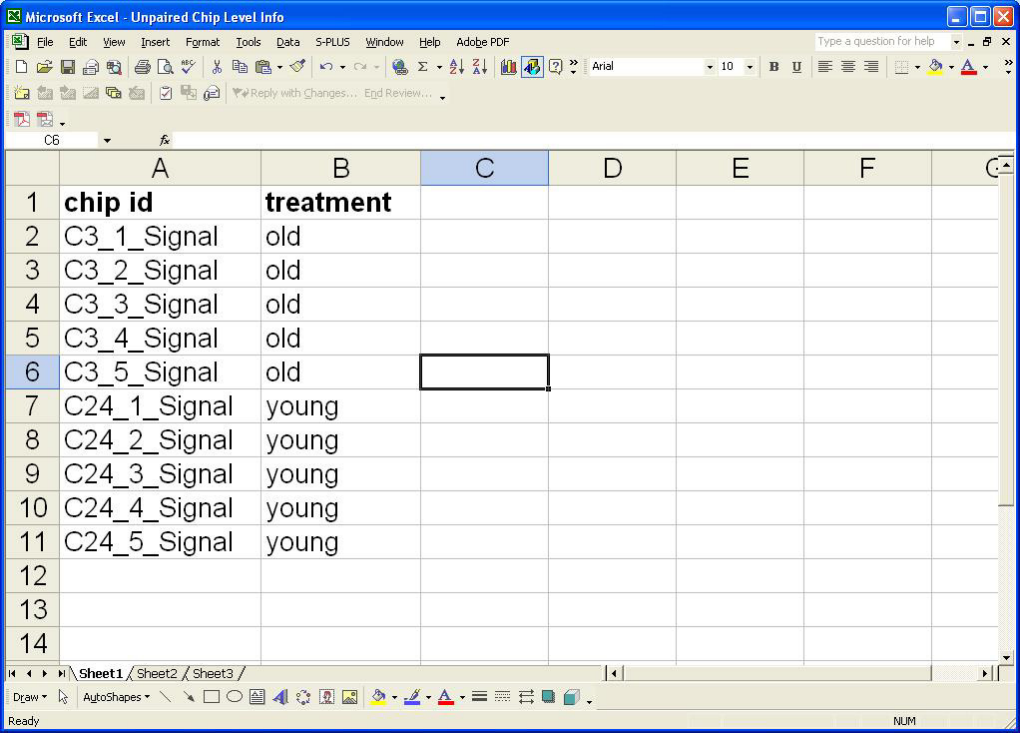
Screenshot of chip level information file in Excel format

Data is imported into HDBStat! using two files, a gene expression data file (Figure 2) and chip level information file (Figure 3), both of which must be Microsoft Excel '97 or more recent format (.xls), or Comma Separated Values (.csv) files [see Additional file 1 and Additional file 2]. The gene expression data file contains the output from the chip image processing software, such as MAS 5.0, Bioconductor, or GenePix. The chip level file contains experimental variables such as treatment, time, experiment, and if appropriate, pairing variables for the chips. Upon import some descriptive statistics are automatically generated about the raw data such as Pearson's correlations between chips, mean, standard deviation, minimum and maximum values of gene expression levels for each chip and displayed in graphical and tabular formats.

### Data preprocessing

Optionally, a normalization and/or transformation method(s) can be applied prior to the primary statistical analyses. Normalization is a procedure intended to remove variability among chips that is unrelated to treatment conditions of interest. HDBStat! offers Chip Mean normalization, which divides each observation by the chip mean, and Quantile-Quantile normalization, which ranks each observation on the chip based on expression value and then converts to the value of a deviation that would be expected from the standard normal distribution based on the observation rank. Quantile-quantile normalization results in data from each chip with a mean of zero and standard deviation of 1.0. Transformation is a process of applying a mathematical function to every observation in a data set in order to better satisfy assumptions of certain statistical models used for analysis. HDBStat! offers three different scales of logarithmic transformation, base-2, base-e, or base-10. Combinations of normalizations and transformations may be selected.

### Quality control

**Figure 4:**
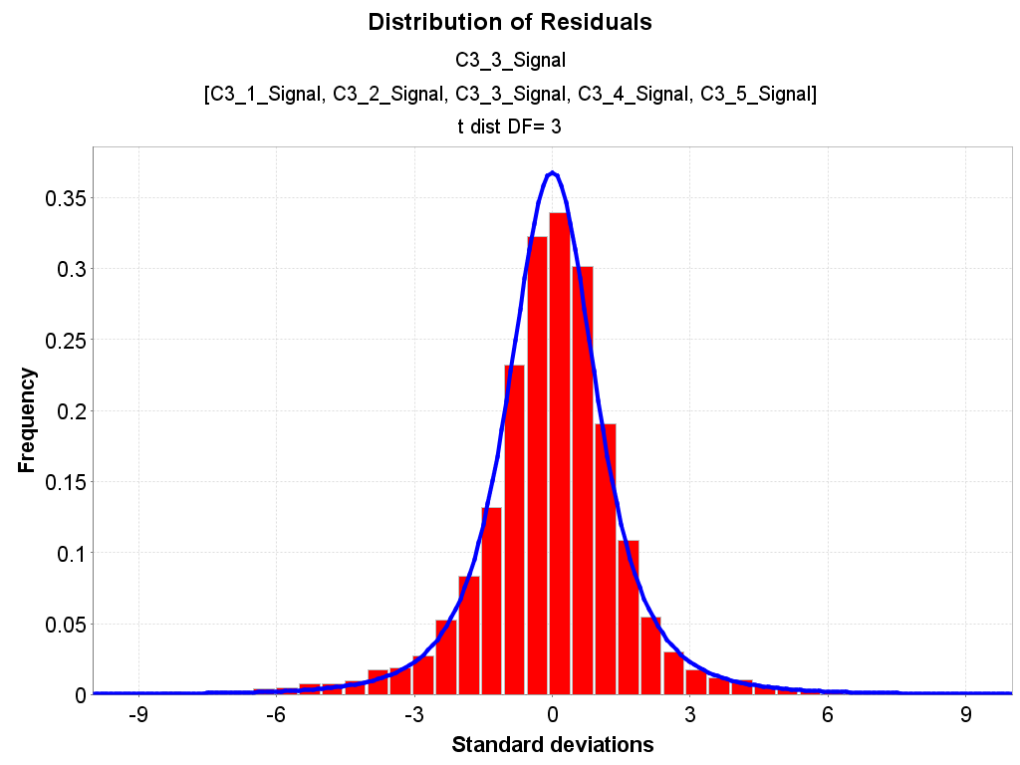
Deleted residuals graph

HDBStat! provides a unique quality control procedure based upon Deleted Residuals (DR). Deleted residuals have traditionally been used in the statistical analysis of data when the number of observations in a group are small or may be influenced by outliers, as in the case in microarrays. In HDBStat!, the deleted residuals for each gene on each chip is calculated by taking the observed value of a gene on a chip subtracting the mean for the gene across all other chips in that group divided by the standard deviation of the mean for the gene across all the other chips in that group. The Probability Density Function (PDF) for the deleted residuals for a gene will follow a Student's t-distribution with n-2 degrees of freedom where n is the number of chips in the treatment group. If we assume that the genes across a chip are independent identically distributed (IID) the distribution of the deleted residuals should approximate a Student's t-distribution with n-2 degrees of freedom. The difference of the observed data from the expected t-distribution is graphically illustrated (Figure 4) and the significance of the difference is tested using a Kolmogorov-Smirnov test. If a chip is significantly different from the t-distribution it may be an indication that the particular chip is an outlier compared with the other chips in the group. Further, the user has the opportunity to remove chip(s) from the analysis and re-analyze the data.

### Hypotheses testing

Currently, HDBStat! performs a series of pair wise comparison tests. Based on the information provided by user in chip level information file, a combination of all possible hypotheses is displayed in the user interface. User must select at least one hypothesis in order to perform two group comparisons.

HDBStat! includes parametric and non-parametric methods for estimating the significance of changes in gene expression between groups. Student's t-test, for which the user can choose an equal-variance t-test, which uses a pooled variance across treatments, or Welch's t-test, which assumes unequal variances between the two treatment groups [11]. Another method based on Chebyshev's inequality, Chebby Checker is extremely robust against departures from normality and equality of variance between treatment groups, but it also has very low power [2]. The Chebby Checker is useful for identifying genes that are almost certainly differentially expressed without considering any statistical assumptions. In addition a bootstrap resampling method [6,8] is implemented. One can either conduct an exact bootstrap (all possible permutations) or a random (used specified number of permutations) bootstrap. The bootstrap procedures implement both pivots and smoothes in order to calculate the significance more accurately. As exact bootstrap is more accurate than random bootstrap, it is preferred for computationally feasible cases, but once the n per groups exceeds 6 it is difficult to implement.

**Figure 5:**
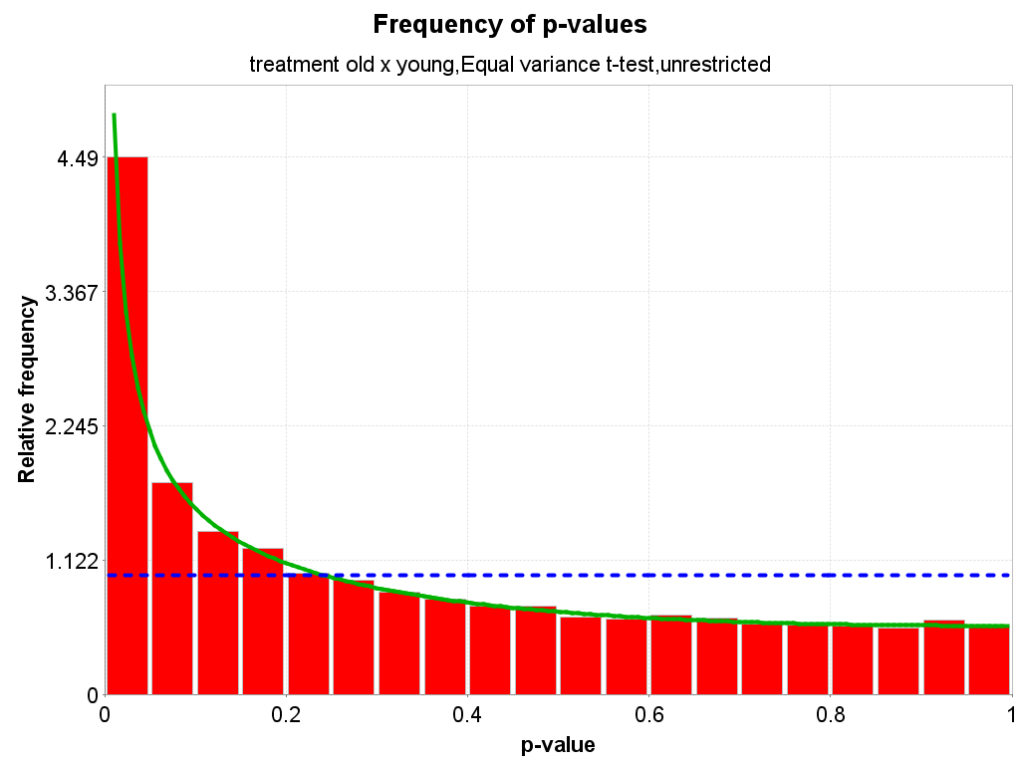
Mix-o-matic graph

Because of the large number of simultaneous hypotheses tested in high dimensional biology experiments, an adjustment for multiple testing is appropriate in order to avoid falsely calling too many genes significant. In HDBStat! several multiplicity control methods are available to be applied to any hypothesis testing method. The available multiplicity control adjustments are Bonferroni [4], Sidak [10], two False Discovery Rate (FDR) estimation methods [3,5], and a method based on a mixture modeling of observed p-values [1], referred to as the "Mix-o-matic" (Figure 5) in the HDBStat! software. The Bonferroni and Sidak methods provide experiment-wise (or Family-wise) type I control. The FDR methods are designed to control the proportion of false positives among all genes declared differentially expressed. The mixture modeling method allows for the Bayesian estimation of the probability that each gene is a false positive or negative and this approach is also conveniently for projecting power estimates for future studies [9].

**Figure 6:**
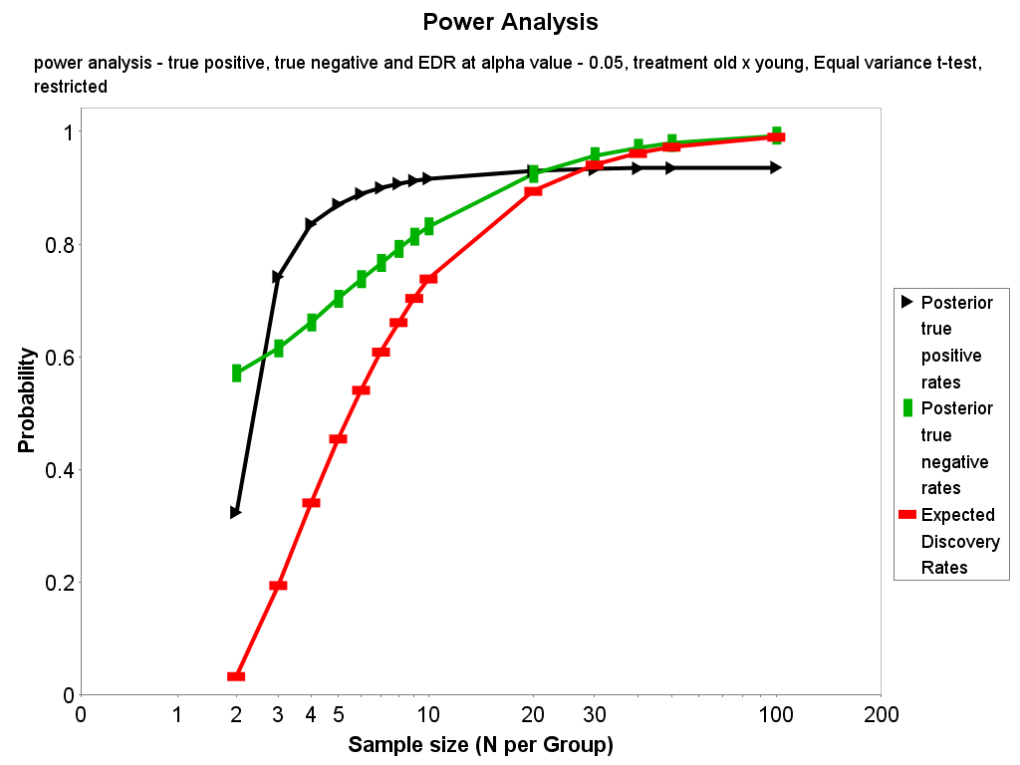
Power analysis graph

For the planning of future studies HDBStat! implements the method of Gadbury et al to extrapolate power from pilot data [9]. HDBStat! allows for the calculation of the expected discovery rate (EDR), posterior true positive, and posterior true negative rates for large and smaller samples sizes than were entered as pilot data. (Figure 6)

If an investigator is interested in empirically comparing the size of the observed differences in gene expression, an Empirical Bayes method is provided to provide shrinkage estimators of the true differences in gene expression [7]. In addition group means and fold changes in expression are calculated and output to results directory specified by the user.

### Programming details

HDBStat! is implemented using the Java programming language using various licensed and open source libraries such as Visual Numerics JMSL, Jakarta POI, Velocity, and JFreeChart. Extensive software testing is performed using JUnit library.

## Results

At the completion of calculating the deleted residuals and analyzing a hypothesis, results are output to a date/time-stamped directory into the user specified directory. Chip level statistics, preprocessed data, deleted residuals, standard outliers, various pair wise comparison tests (Table 1), mix-o-matic and power analysis results are output in the form of Excel CSV files. Graphs generated from chip level statistics, deleted residuals, mix-o-matic and power analysis results are output in .png format image files. HDBStat! also generates a HTML file that provides a summary of the analysis including the hypotheses tested, chips in each group. This mechanism of outputting results provides the user an opportunity to view quality control results and modify hypotheses, preprocessing methods, and/or chip selections before proceeding to the next step.

## Discussion

The goal of HDBStat! is to help researchers analyze microarray data to extract valid inferences, estimates and interpretations via a flexible and user-friendly graphical interface. It allows the user to skip preprocessing and quality control methods by simply not selecting those methods. After previewing the preliminary results of raw data, preprocessed data or deleted residuals, user has flexibility to drop a chip by simply un-checking checkbox in the user interface. This feature allows the user to design any number of possible comparisons while the analysis is in progress.

To assist novice users with using HDBStat!, video clips demonstrating how to analyze paired and unpaired data, examples of how to set up input files for paired and unpaired data analyses, screen shots, and FAQ are available on our website. A detailed description of methods as well as additional explanations of the output files in this software is also available in a PDF format on our website.

Additional statistical methods and features are added on an ongoing basis. Support for data import from a text file and results output to a text file will be available for large data sets. In the current version, only single channel or common reference design microarray data can be analyzed using two group comparisons. In the near future, we will add the capability to analyze two channel data and support for ANOVA, and GLM.

There are many software programs available to analyze microarray data, each offering various features and functions. In Table 2, we have compared the features and functions of HDBStat! to SAM, BRB Array Tools and TM4.

## Availability and requirements

System requirements for an end-user are the Java Runtime Environment (JRE 1.4.2 or higher), at least 256 MB RAM and 25 MB hard disk space. Using Java Web Start technology, HDBStat! can be easily downloaded from our website at .

## Authors' contributions

JWE, JPLB, KK, GPP wrote the implementation specification documents for various methods. GLG and DBA developed mix-o-matic method, developed prototype implementation in S-Plus and wrote the implementation specification document. GPP and JWE developed the deleted residuals approach. DBA and JWE developed prototype implementation of Empirical Bayes Estimates. JWE and PT developed a prototype implementation of statistical methods in SAS. JW, PT, VS and TM implemented and tested the java code. JWE, PT, JW, SOZ, GPP, VS, TM and AP tested the software. GPP and JWE directed the content of HDBStat! All authors read and approved the manuscript.

## Figures and Tables

**Table 1:** Hypothesis testing results

**Probe**	**Mean (group1, Raw data)**	**Mean (group2, Raw data)**	**Fold change**	**t (Equal variance t-test)**	**p-value (Equal variance t-test)**	**PTP, unrestricted (Equal variance t-test)**
AFFX-MurIL2_at	105.16	200.34	1.905097	-0.81193	0.440318	0.226179

AFFX-MurIL10_at	176.52	253.58	1.436551	-0.49777	0.632041	0.194216

AFFX-MurIL4_at	146.56	197.86	1.350027	-0.05477	0.957668	0.147303

AFFX-MurFAS_at	670.06	487.42	0.727427	2.264698	0.05333	0.32061

AFFX-BioB-5_at	4074.98	5165.36	1.267579	-1.10225	0.302405	0.251946

AFFX-BioB-M_at	10258.4	12376.04	1.20643	-0.85246	0.418745	0.230018

AFFX-BioB-3_at	5600.76	6784.9	1.211425	-0.82656	0.432446	0.227573

AFFX-BioC-5_at	15688.4	17406.88	1.109538	-0.23558	0.819676	0.16602

AFFX-BioC-3_at	11812.54	13288.74	1.124969	-0.2571	0.803593	0.168321

AFFX-BioDn-3_at	65025.2	68451.72	1.052695	0.467328	0.65273	0.190965

AFFX-CreX-5_at	124944.9	152682.8	1.222001	-0.39488	0.70325	0.183174

AFFX-CreX-3_at	166352	192790.6	1.158932	-0.26323	0.799024	0.168979

AFFX-BioB-5_st	611.6	677.8	1.108241	0.280987	0.785851	0.170886

AFFX-BioB-M_st	556.26	779.84	1.401934	-1.1822	0.27107	0.25832

AFFX-BioB-3_st	195.62	359.32	1.836827	-1.51174	0.169049	0.281499
AFFX-BioC-5_st	114.36	183.8	1.607205	-2.17372	0.061461	0.316596

AFFX-BioC-3_st	57.36	94.86	1.653766	-0.62822	0.547374	0.2079

AFFX-BioDn-5_st	1981.88	2327.5	1.17439	-0.99146	0.350498	0.242595

AFFX-BioDn-3_st	1487.74	2265.06	1.522484	-2.01951	0.078118	0.309434

AFFX-CreX-5_st	950.56	1221.04	1.284548	-1.42444	0.192137	0.275814

AFFX-CreX-3_st	2610.3	3429.5	1.313834	-1.21894	0.257585	0.261147

AFFX-DapX-5_at	204.42	267.12	1.306721	0.187522	0.85592	0.16092

AFFX-DapX-M_at	299.3	347.62	1.161443	0.119799	0.907597	0.153866

AFFX-DapX-3_at	192.64	95.82	0.497404	1.5611	0.157123	0.284581

AFFX-LysX-5_at	132.46	115.9	0.874981	0.445594	0.667702	0.188634

AFFX-LysX-M_at	174.16	204.14	1.172141	-0.09641	0.92557	0.151477

AFFX-LysX-3_at	47.8	167.94	3.513389	-1.2216	0.256632	0.261348

AFFX-PheX-5_at	33.04	55.5	1.679782	-2.04285	0.07534	0.310549

AFFX-PheX-M_at	40.64	29.66	0.729823	1.223746	0.255865	0.261511

AFFX-PheX-3_at	497.66	217.12	0.436282	2.826877	0.022257	0.342932

AFFX-ThrX-5_at	92.06	184.74	2.006735	-0.26462	0.797994	0.169127

AFFX-ThrX-M_at	225.32	272.46	1.209214	-0.33299	0.747704	0.17649

AFFX-ThrX-3_at	114.54	95.08	0.830103	-0.17193	0.86776	0.15928

AFFX-TrpnX-5_at	73	96.68	1.324384	-0.82529	0.433126	0.227452

AFFX-TrpnX-M_at	30.12	37.74	1.252988	-0.31889	0.757976	0.174969

**Table 2:** Comparison of HDBStat! with other software packages. All these software packages are still in active development and new functions will undoubtedly be added over time.

	**HDBStat!**	**SAM**	**BRB-Array Tools 3.2.2**	**TM4**
Two color data handling	Common reference and balanced block designs	No	Yes	Yes

Database	No	No	No	Yes

Ratio Statistics	Yes	No	Yes	Yes

Normalization	Yes	Yes	Yes	Yes

Max number of arrays	No limit	255	249	No limit

Discriminate Analysis	No	No	Yes	Yes

ANOVA	Yes	No	Yes	Yes

Bootstrapping	Yes	Yes	Yes	Yes

Non-normal and heteroskedastic data handling	Yes	Yes	Via normalization	Via normalization

Non-parametric statistics	Yes	No	Yes	No

Cluster analysis	No	No	Yes	Yes

FDR (number)	8	1	2	1

Family Wise Error rate corrections	2	0	1	1

Quality Control	Yes	No	No	Yes

Power Analysis	Yes	No	No	No

Automatic report generation	Yes	No	Yes	No

Gene Class testing	No	No	Yes	No

Automatic Annotation	No	Link out	Yes	No

Platform	Single program implemented in Java & available via Java Web Start technology	Microsoft Excel Add-in	Microsoft Excel Add-in	4 separate programs, 3 of which implemented in java & 1 in C++
